# Preparation of baicalin-loaded ligand-modified nanoparticles for nose-to-brain delivery for neuroprotection in cerebral ischemia

**DOI:** 10.1080/10717544.2022.2064564

**Published:** 2022-04-25

**Authors:** Xinxin Li, Shuling Li, Chun Ma, Tieshu Li, Lihua Yang

**Affiliations:** a College of Chinese Medicine, Changchun University of Chinese Medicine, ChangChun, China; b Affiliated Hospital of Changchun University of Chinese Medicine, ChangChun, China

**Keywords:** Nose-to-brain, rabies virus glycoprotein 29, nanoparticles, cerebral ischemia, baicalin

## Abstract

Neuroprotection in cerebral ischemia (CI) has received increasing attention. However, efficient delivery of therapeutic agents to the brain remains a major challenge due to the complex environment of the brain. Nose-to-brain-based delivery is a promising approach. Here, we optimized a nanocarrier formulation of neuroprotective agents that can be used for nose-to-brain delivery by obtaining RVG29 peptide-modified polyethylene glycol–polylactic acid-co-glycolic acid nanoparticles (PEG–PLGA RNPs) that have physicochemical properties that lead to stable and sustained drug release and thereby improve the bioavailability of neuroprotective agents. The brain-targeting ability of PEG–PLGA RNPs administered through nasal inhalation was verified in a rat model of CI. It was found that delivery to the whole brain can be achieved with little delivery to the peripheral circulation. Baicalin (BA) was selected as the neuroprotective agent for delivery. After intranasal administration of BA–PEG–PLGA RNPs, the neurological dysfunction of rats with ischemic brain injury was significantly alleviated, the cerebral infarction area was reduced, and nerve trauma and swelling were relieved. Furthermore, it was demonstrated that the neuroprotective effects of BA in a rat model of CI may be mediated by inhibition of inflammation and alleviation of oxidative stress. The immunohistochemical results obtained after treatment with nanoparticles loaded with BA showed that Nrf2/HO-1 was activated in the area in which ischemic brain damage had occurred and that its expression was significantly higher in the group treated with BA–PEG–PLGA RNPs than in the other groups. The ELISA results showed that the levels of IL-1β, IL-6, and TNF-α were abnormally increased in the serum of rats with cerebral ischemia. After treatment with BA-loaded nanoparticles, IL-1β, IL-6, and TNF-α levels decreased significantly. Oxidative stress was alleviated; the levels of glutathione and superoxide dismutase increased; and the levels of reactive oxygen species and malondialdehyde decreased, in animals to which BA–PEG–PLGA RNPs were delivered by intranasal inhalation. In conclusion, BA–PEG–PLGA RNPs can effectively deliver BA to rats and thereby exert neuroprotective effects against CI.

## Graphical Abstract

**Figure:**
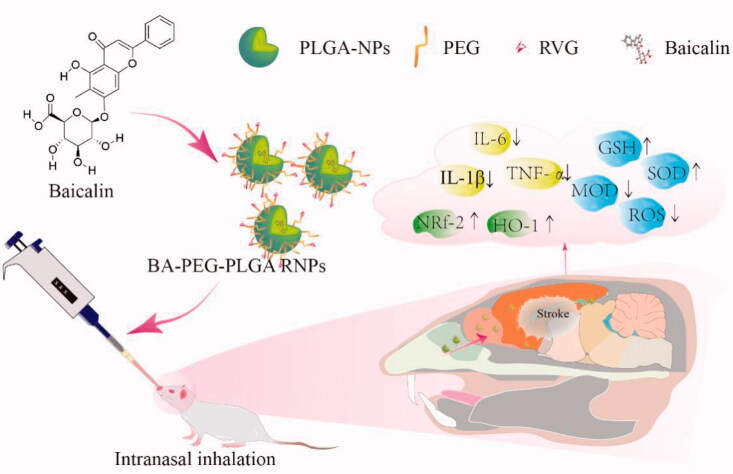

## Introduction

Stroke seriously threatens human health and affects quality of life. Cerebral ischemic stroke accounts for approximately 70% of all stroke cases (Ma et al., 2020), and nerve damage that develops after cerebral ischemia (CI) is usually irreversible. One of the reasons for this irreversible damage is oxidative stress damage, which leads to an increase in reactive oxygen species (ROS) and a decrease in glutathione and superoxide dismutase, resulting in neuronal damage and even death (Md et al., 2019). Currently, thrombolytic therapy for ischemic stroke is a standard treatment that uses antithrombotic drugs such as urokinase and alteplase to improve cerebral blood circulation (Powers et al., 2019). However, the application of this treatment modality is limited by a short therapeutic window and a high risk of bleeding complications. In addition, neuronal damage caused by CI has received increasing attention, and neuroprotective agents such as edaravone are used to reduce neuronal death caused by ischemia and reperfusion injury. However, most neuroprotective agents used in the treatment of ischemic stroke have drawbacks such as limited half-life, potential toxicity, poor tissue specificity, and others. In addition, the existence of the blood–brain barrier (BBB) also limits the application of traditional drug therapy, and it is difficult to achieve effective drug accumulation in lesion areas. Therefore, exploration of new therapeutic strategies for the treatment of ischemic stroke is urgently needed. Traditional Chinese medicine has been used in the clinical treatment of cerebrovascular diseases in China, South Korea, Japan, and other Asian countries (Sun et al., 2015); however, due to the existence of the BBB, it is necessary to develop methods that can effectively deliver these drugs to the brain.

Baicalin (BA) usually refers to 7-D-glucuronic acid 5,6-dihydroxyflavone, a flavonoid extracted from the rhizome of *Scutellaria baicalensis*. BA exerts anti-inflammatory and antioxidant pharmacological effects by reducing the generation of ROS, thereby upregulating the expression of nuclear factor erythroid 2-related factor 2 (Nrf2) and heme oxygenase 1 (HO-1), reducing damage to brain tissue, and exerting neuroprotective effects in rats that have been subjected to CI (Huang et al., 2021). BA, one of the main components of Qingkailing injection, is a Chinese patent medicine that has been approved by the State Food and Drug Administration for the treatment of ischemic stroke and is widely used in clinical practice (Ma et al., 2020). However, its clinical application has certain limitations. In particular, it has poor solubility in water (Xiang et al., 2020) and this not only affects the way in which it is prepared and administered but also restricts its bioavailability *in vivo*. To improve the solubility of BA, researchers have developed a variety of pharmaceutical formulations, including nanoemulsions and liposomes, and have attempted to load BA into encapsulating materials such as cyclodextrin and polyethylene glycol–polylactic acid-co-glycolic acid (PEG–PLGA). Although these formulations help improve the solubility of drugs in body fluids, their improvement might increase the efficiency of brain targeting of drugs that are used in the treatment of brain diseases.

Improving the bioavailability of medicine for the treatment of brain diseases could be achieved by transforming the method of drug administration. Nasal inhalation is a promising way to deliver drugs to the brain rapidly and effectively because it can bypass the BBB. Intranasal inhalation of medicine could enhance the bioavailability of the medicine in the brain by increasing its concentration in the cerebrospinal fluid. It has been reported that the concentration of BA in brain tissue was significantly higher after nose-to-brain drug delivery (NBDD) than after intravenous administration, an effect that would be beneficial in the treatment of diseases of the central nervous system (Long et al., 2020). However, it is still necessary to consider the problem of how to increase the efficiency of brain targeting when a drug enters the brain from the nose because there is still a possibility that the drug may enter the blood and subsequently be distributed through the systemic circulation. At present, nanoparticles (NPs) have made some progress in the treatment of neurodegenerative diseases. Some studies have shown that the preparation of drugs into NPs and the nasal-brain administration method shows obvious advantages over conventional drug administration, improving the biodistributes in brain tissue, improving pharmacodynamics, and providing neuroprotection (Md et al., 2019; Bhattamisra et al., 2020). Our previous work demonstrated the potential of intranasal delivery of PEG–PLGA NPs modified with RVG29 in the treatment of brain diseases. We expect to improve the application of BA in the treatment of brain diseases by improving the NP formulation and by applying NPs modified with brain-targeting ligands using NBDD.

**Scheme 1.**
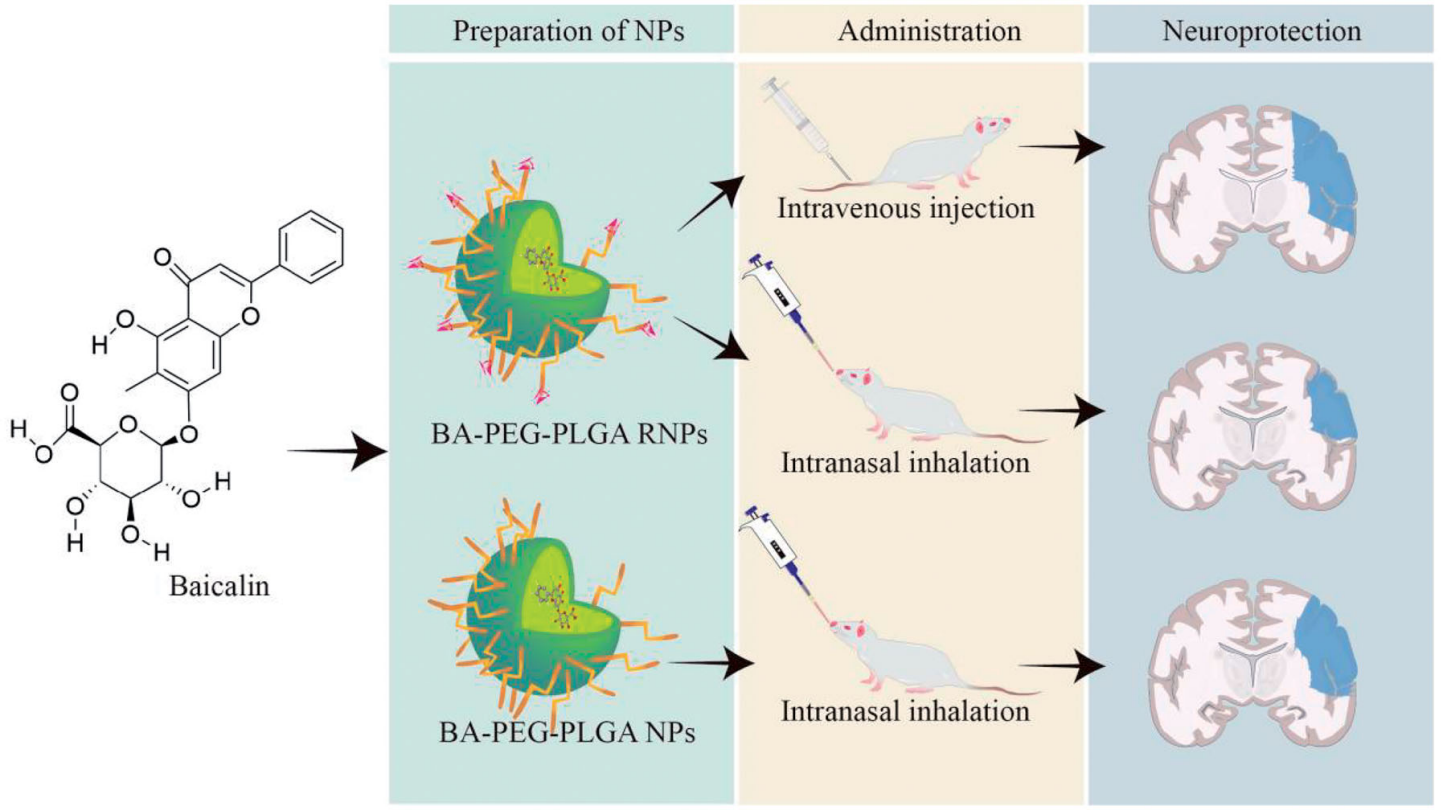
Preparation of ligand-modified baicalin-loaded nanoparticles for neuroprotection in the treatment of ischemic brain injury via nose-to-brain delivery.

Here, we attempted to use RVG29-modified PEG–PLGA NPs to carry BA and to achieve brain targeting of ligand-modified NPs by nasal inhalation. The *in vivo* brain-targeting ability of the ligand-modified NP preparations was assessed by studying the distribution of DiR preparations in rats. At the same time, the feasibility of using the optimized BA-loaded nanopreparations to treat brain diseases was studied using a rat model of CI established by the MCAO method. Herein, we present a method to improve the *in vivo* application of BA and thereby establish a platform for the development of new natural medicines for clinical applications. More importantly, an NBDD-based NP preparation process was established, laying the foundation for the development of new natural medicines for the treatment of ischemic brain injury (Scheme 1).

## Methods

### Materials

PEG–PLGA and maleimide-poly(ethylene glycol)-poly(lactic co-glycolic acid) were purchased from Daigang Biomaterials Co., Ltd. (Jinan, China). BA (purity >95%) was purchased from Shanghai Ryron Biological Technology Co. (Shanghai, China). DiR (1,1′-dioctadecyl-3,3,3′,3′-tetramethylindotriccarbocyanine iodide) was purchased from Sigma–Aldrich (St. Louis, MO, USA). Pentobarbital sodium was purchased from Sigma. 2,3,5-Triphenyltetrazolium chloride (TTC) was purchased from Sigma. Hematoxylin and eosin (HE) were both purchased from Sigma. RVG29-Cys-peptide was purchased from Changchun Xinjinji Biotechnology Co., Ltd (Changchun, China). All chemical reagents used in the experiments were of analytical grade unless otherwise specified.

### Preparation and characterization of BA-loaded PEG–PLGA NPs

#### Preparation of BA-loaded PEG–PLGA NPs

BA-loaded PEG-PLGA NPs were prepared by the double emulsification technique according to previously published methods with some modifications. Two milligrams of BA were dissolved in 1 mL of phosphate-buffered saline (PBS) and mixed with 2 mg of male-PEG-PLGA dissolved in 2 mL of dichloromethane as the oil phase, and the mixture was sonicated for 40 s to form a primary emulsion. The primary emulsion was added to 25 mL of a solution containing 0.5% or 1% (w/v) sodium cholate (SC) and further solidified into NPs. The organic solvent was removed by rotary evaporation. DiR-labeled NPs were prepared in the same way.

The nanoprecipitation method for preparing BA-loaded NPs was performed as described in previous studies, with some modifications (Lee et al., 2010; Said-Elbahr et al., 2016). Two milliliters of dimethylformamide, in which 2 mg of BA and 15 mg of PEG–PLGA polymer (Hao et al., 2020) were dissolved, were added dropwise using a syringe to 15 mL of distilled water containing 0% or 1% poloxamer P407, and the mixture was stirred at room temperature for 6 h to allow the organic solvent to evaporate completely.

#### Characterization of brain-targeting ligand-modified NPs

RVG29 peptide-modified NPs were prepared according to the method described previously (Hao et al., 2020). PEG–PLGA NPs were incubated with excess RVG29 peptide for 8 h at room temperature with rotation. The resulting PEG–PLGA RNPs were collected by centrifugation at 12,000 × *g* for 15 min. The amount of RVG29 incorporated into the NPs was determined using a Tecan Infinite 200 PRO NanoQuant (Switzerland) and calculated by measuring the total amount of RVG29 and the residual amount of RVG29 in the supernatant. The morphology of the NPs was characterized by transmission electron microscopy (TEM, Tecnai Spirit Biotwin, Japan), and the hydrodynamic diameter (DLS) and zeta potential of the particles were characterized using a dynamic light scattering particle analyzer (Zetasizer Nano ZS, UK). Trehalose (5%, w/v) was added to the NP solution as a cryoprotectant. Then NPs were frozen, lyophilized, and stored at −20 °C.

### Evaluation of the encapsulation efficiency and *in vitro* release of BA

The BA content of the supernatant was determined using high-pressure liquid chromatography (HPLC) (Ashraf et al., 2018). BC was separated on a C18 (Hypersil ODS, Thermo Scientific) column (250 mm × 4.6 mm; 5 μm) in a mobile phase of methanol/water; the effluent was monitored at 279 nm (Han et al. 2019). The encapsulation efficiency (EE) of PEG-PLGA NPs was calculated as follows:

EE(%)=total feeding amount of baicalin−free mass of baicalin/total feeding amount of baicalin

The amount of BA released from each of the selected formulations was determined. One milliliter of each nanoformulation containing BA-loaded PEG–PLGA RNPs was placed in a dialysis bag (dialysis membrane, MWCO = 12–14 kDa), and the dialysis bag was incubated at 37 °C in 200 mL of phosphate buffer (pH 6.5) as the release medium. Samples of the release medium were collected at 0.5, 1, 2, 4, 8, 12, 24, 36, and 48 h, and the amount of BA released was measured using HPLC as previously described. At each sample collection, the system was replenished by the addition of an equivalent volume of fresh PBS. The BA–PEG–PLGA NPs stored at −20 °C for 0, 7, 14, 28, 60, and 90 days were tested for encapsulation efficiency to verify the drug release stability of BA–PEG–PLGA NPs.

### Analysis of the intracerebral distribution of NPs

Fluorescence distribution tracking in rat brain was performed using DiR-labeled PEG–PLGA NPs and PEG–PLGA RNPs. For intranasal administration, two types of fresh solutions of NPs were alternately applied to both nostrils of rats using a pipette. For intravenous administration, the same two types of NPs, PEG–PLGA NPs, and PEG–PLGA RNPs, containing the same equivalent amounts of BA were injected into the tail veins of rats. At 30 and 120 min after injection or intranasal administration, the animals were decapitated, their blood was collected, and a craniotomy was rapidly performed. The brains of the animals were immediately dissected into their major anatomical regions (olfactory bulb, cortex, striatum, midbrain, hippocampus, and cerebellum) using fine-tipped forceps. Surface blood was removed by washing with normal saline.

Homogenates of brain tissue from each group were added to a 96-well plate in which each well contained 10 µL of DMSO. The fluorescence intensities of the samples were read at 750/780 nm (excitation/emission wavelengths) on a plate spectrophotometer (Tecan Infinite 2000). All samples were taken in triplicate, and the units were converted to ng DiR/g (Cook et al., 2015; Chung et al., 2020). Measurement of fluorescence in other peripheral tissues and organs was conducted using basically the same method used for brain tissue.

### Experimental animals and groups

#### Animals

Male rats (280–320 g) were housed in transparent cages in a clean environment under controlled temperature, moderate conditions under an alternating light/dark cycle, and fed standard solid chow and water ad libitum. All procedures complied with the relevant animal ethics regulations.

#### Grouping

The rats were randomly divided into six groups: a sham group, a model control group, a BA buffer group, a BA–PEG–PLGA NP group, a BA–PEG–PLGA RNP group, and a BA–PEG–PLGA RNP with intravenous administration group. The animals were given corresponding treatments 3 days before modeling.

### Drug administration

A total of 0.2 mL of BA–PEG–PLGA NPs and BA–PEG–PLGA RNPs (containing 9.00 mg·mL^−1^ BA) and 0.2 mL of PBS buffer containing BA at 9.00 mg mL^−1^ were prepared for intranasal administration in the BA–PEG–PLGA NP group, the BA–PEG–PLGA RNP group, and the BA buffer group. The rats in the intravenous injection group received intravenous injections of 0.8 mL of BA–PEG–PLGA RNPs (2.25 mg·mL^−1^ BA, prepared with normal saline).

### Establishment of MCAO model of cerebral ischemia

A rat model of focal cerebral ischemia (MCAO) was created using the modified line embolus method. Anesthesia was performed by intraperitoneal injection of 3% pentobarbital sodium. The common carotid, internal and external carotid arteries were exposed. The common carotid artery was clipped, the external carotid artery was ligated, and a small incision was made at the bifurcation of the external carotid artery and the internal carotid artery near the external carotid artery. When the insertion length of the suture was approximately 17–18 mm, the suture was fixed, the common carotid artery was released, and the suture was secured. After 2 h, two-thirds of the suture was pulled out. After 24 h, behavioral tests were performed, blood samples were collected, the rats were sacrificed, and tissue samples were collected. Part of the brain tissue was embedded in paraffin and sectioned for later use.

### Neurological assessment

Evaluation of neurological function was performed before the rats in each group were sacrificed. The Zea Longa score (Longa et al., 1989) was first used to score each group, and the neurological deficit of the rats was judged by their behavioral performance. Then the balance beam test (Zhong et al., 2019) was performed at the same time. The rats were trained to pass through a beam with a length of 1.75 m, a width of 1.9 cm, and a height of 0.7 m, a six-point scale was adopted in the test. Their motor coordination and the balance control of their forelimbs and hindlimbs were evaluated based on their behavioral responses as a means of assessing the recovery of nerve function in the postischemic rats in each group.

### Assessment of infarct volume

The cerebral infarction volumes in individual rats were measured by the TTC staining method. Each rat was anesthetized, its cerebellum and olfactory bulbs were removed, and the remaining brain tissue was sliced coronally into 2-mm slices using a razor blade. Each slice was placed in a water bath at 37 °C for 30 min while protected from light. The container was shaken gently every 5 min to ensure complete staining. After staining, the brain slices were removed and fixed overnight in 10% paraformaldehyde. Sections were photographed and analyzed using ImageJ software.

### Nerve TEM

The rats in each group were anesthetized and perfused with 2% paraformaldehyde and 2% glutaraldehyde. Tissue samples were removed and fixed in 3% glutaraldehyde for 2 h. The samples were then precooled and placed in 0.1 mol·L^−1^ phosphate buffer, and ultrathin sections were cut. Ultrastructural changes in neurovascular units were observed using TEM (Hitachi, Japan).

### HE staining

The morphological characteristics of cerebral infarct tissue in rats were observed after HE staining. Paraffin sections of brain tissue were dewaxed and stained with hematoxylin solution. The sections were then differentiated with 1% hydrochloric acid ethanol, washed with water, and stained with eosin solution. After dehydration, the sections were cleared twice in xylene twice and mounted. HE staining was performed, and the sections were observed under light microscopy.

### Immunohistochemistry

The deparaffinized sections were incubated with 3% hydrogen peroxide solution at room temperature for 15 min, and washed with PBS (pH 7.4). 3% BSA was used for blocking for 30 min. The sections were incubated overnight at 4 °C with primary antibodies Nrf2 and HO-1 (Bioworld technology co., Ltd, Nanjing, China). Before incubating with biotinylated goat anti-rabbit IgG (Abeam, UK), the slides were rinsed three times with PBS (pH 7.4) for 5 min each, then they were placed in PBS for destaining. After the sections were slightly dried, DAB was used to develop the color, following by counterstaining with Harris hematoxylin. The slides were washed with tap water, returned to blue, and they were rinsed again with running water. The samples were then dehydrated, mounted, and examined under a microscope. Five fields of view in each of the three slices from each rat were randomly selected for image processing. Cells with brownish-yellow cytoplasm were counted as positive cells. The numbers of Nrf2 and HO-1 immunoreactive cells in the ischemic hippocampus were calculated using Image J.

### Measurement of the levels of inflammatory factors

The levels of IL-1β, IL-6, and tumor necrosis factor-α (TNF-α) in rat serum were measured by enzyme-linked immunosorbent assay (ELISA). After the serum to be tested was removed, it was fully thawed and centrifuged again. An ELISA kit (Nanjing Jiancheng Bioengineering Institute, Nanjing, China) was used to set and record blank wells, standard product wells and test sample wells as needed. After addition of the reagent according to the instructions, the absorbance of the samples at 450 nm was measured using a microplate reader (BioTek Instruments, Inc., USA), and the results were recorded.

### Oxidative stress detection

Brain tissues of rats in each group were collected, ROS generation in the ischemic brain tissues was measured using a ROS assay kit (Nanjing Jiancheng Bioengineering Institute, Nanjing, China), and the final fluorescence value was recorded. An malondialdehyde (MDA) assay kit, a reduced glutathione (GSH) assay kit, and a total superoxide dismutase (SOD) assay kit, all from Nanjing Jiancheng Bioengineering Institute, were used to measure the levels of various factors in ischemic brain tissue. The absorbance values of the samples were measured using a microplate reader.

### Statistical analysis

All data are presented as the mean and standard deviation (mean ± SD). Statistical analysis was performed using GraphPad Prism 9 software. Data analysis was verified by one-way ANOVA and repeated Tukey’s test. *p* < .05 indicates a significant difference.

## Results

### Preparation and characterization of PEG–PLGA NPs

**Figure 1.**
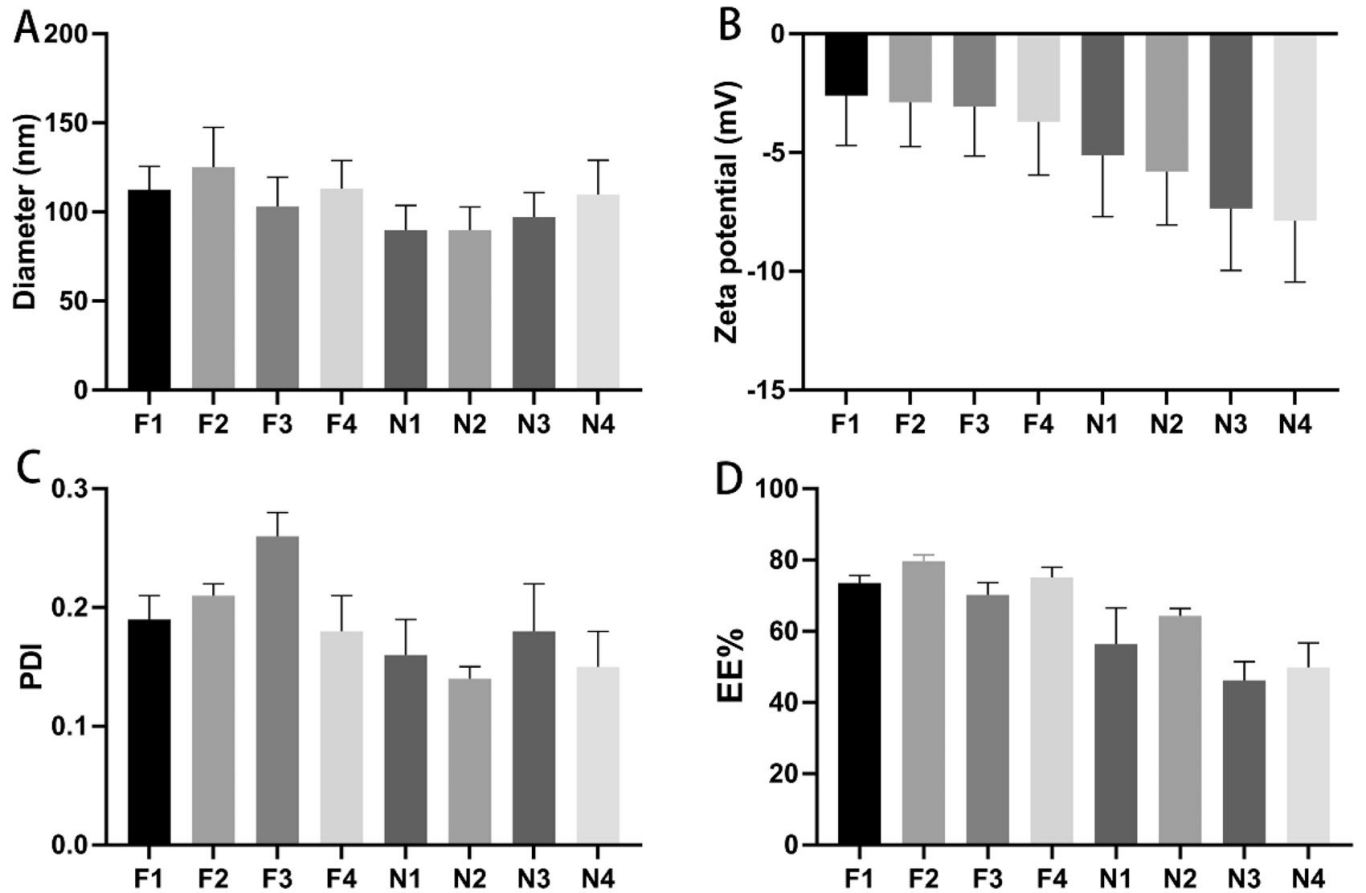
Characterization of different BA-loaded PEG–PLGA nanoparticles (mean ± SD, *n* = 3). (A) Particle sizes of PEG–PLGA nanoparticles; (B) zeta potentials of PEG–PLGA nanoparticles; (C) PDIs of PEG–PLGA nanoparticles; (D) encapsulation efficiencies of PEG–PLGA nanoparticles.

Eight formulations of PEG–PLGA NPs encapsulated with BA were successfully prepared (see Table 1 for formulations). The particle sizes, potentials, PDIs, and encapsulation efficiencies of the NPs are shown in Figure 1. The average particle size of the prepared PEG–PLGA NPs ranged from 89 to 130 nm. The surface charges of all NPs were characterized. The zeta potentials of these eight formulations are between −2 and −10 mV, and their PDI values are approximately 0.10 to 0.30. The encapsulation efficiency of BA of NPs (F1–F4) prepared using the emulsion solvent evaporation technique was higher than that of NPs prepared using the nanoprecipitation technique (N1–N4). The F2 formulation achieved the highest encapsulation efficiency of 79.6%. The properties of the PEG–PLGA NPs are shown in Figure 1. Formulas F4 and N2 were selected based on the hydrodynamic diameters, zeta potentials, PDIs, and encapsulation efficiencies of the NPs and used to further prepare BA-loaded RVG29 peptide-modified BA–PEG–PLGA RNPs. As shown in the Supplementary Table S2, the encapsulation efficiency of BA–PEG–PLGA NPs stored at −20 °C decreased slightly after 3 months, indicating that the NPs we prepared are stable.

**Table 1.** Compositions of baicalin-loaded PEG-PLGA nanoparticle formulations.

Formulation number	PEG–PLGA	Poloxamer P407	Sodium cholate
F1	PEG2k–PLGA50/50 18k	–	0.5%
F2	PEG4k–PLGA50/50 35k	–	0.5%
F3	PEG2k–PLGA50/50 18k	–	1%
F4	PEG4k–PLGA50/50 35k	–	1%
N1	PEG2k–PLGA50/50 18k	0.1%	–
N2	PEG4k–PLGA50/50 35k	0.1%	–
N3	PEG2k–PLGA50/50 18k	–	–
N4	PEG4k–PLGA50/50 35k	–	–

### TEM of PEG–PLGA NPs modified with the brain-targeting ligand RVG29

**Figure 2.**
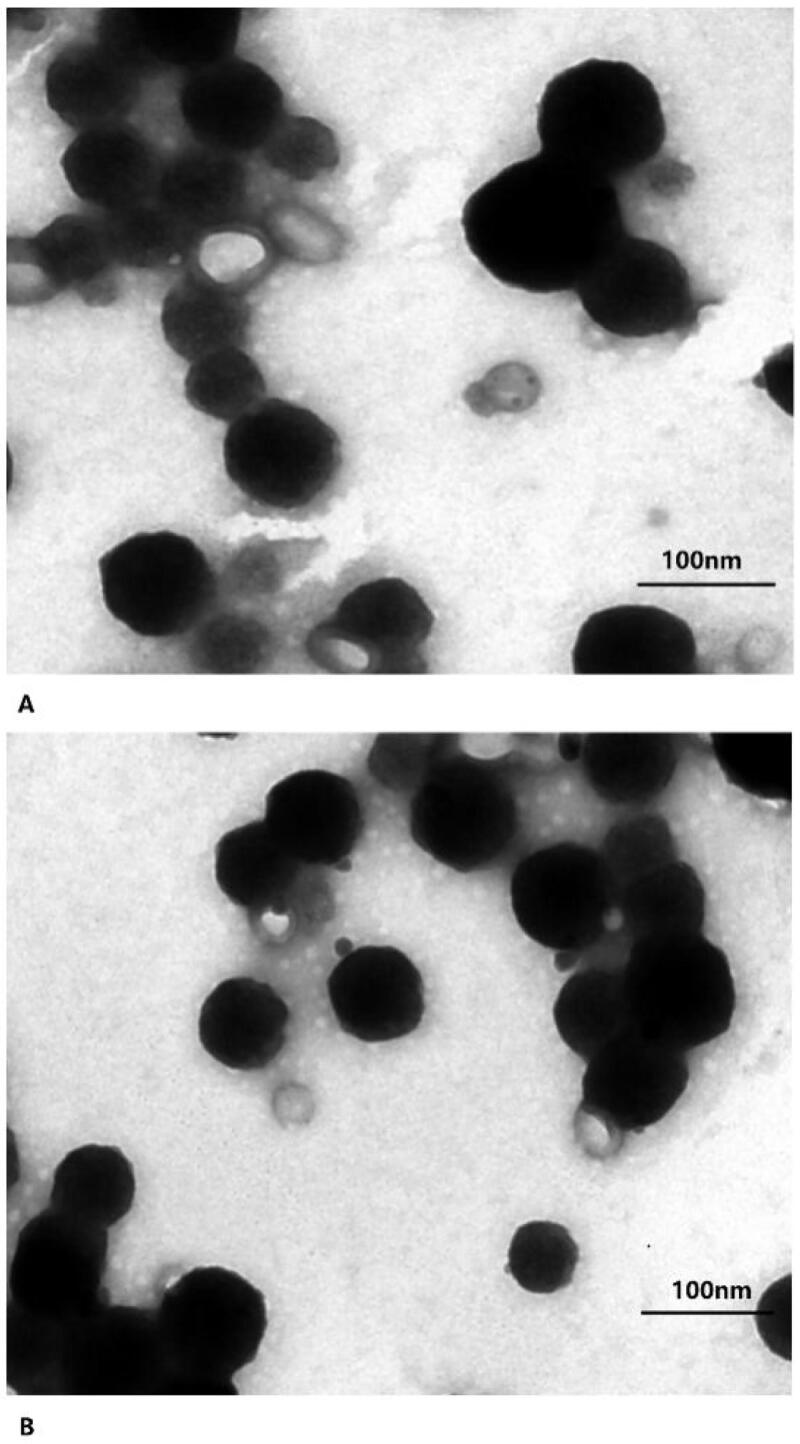
Representative TEM of BA–PEG–PLGA RNPs prepared using the F4 (A) and N2 (B) formulations.

The morphology of the NPs was characterized by TEM. Figure 2 shows representative TEM images of BA–PEG–PLGA RNPs prepared using the F4 and N2 formulations. The images show that the NPs prepared by the nanoprecipitation method are lighter in color than those prepared by the emulsion evaporation method. The poloxamer coating is evident around the particles prepared according to the N2 formulation. The NPs prepared by both methods exhibited spherical core-shell structures.

### Evaluation of the *in vitro* release of BA

**Figure 3.**
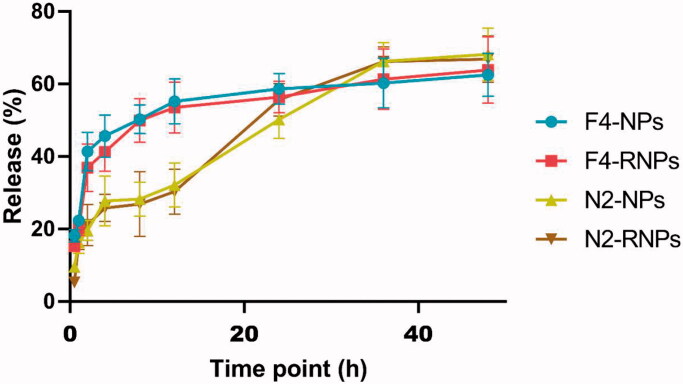
Cumulative percent release over 48 h of BA from BA–PEG–PLGA RNPs and BA–PEG–PLGA NPs prepared using the F4 and N2 formulations (*n* = 3).

Figure 3 shows the release profiles of BA–PEG–PLGA RNPs prepared with formulations F4 and N2. The release of BA from F4–BA–PEG–PLGA RNPs modified with RVG29 over 48 h was similar to that of its release from unmodified BA–PEG–PLGA NPs. NPs prepared using the F4 formulation exhibited a significant burst release at 4 h followed by a controlled release.

### Rvg29-mediated brain targeting

**Figure 4.**
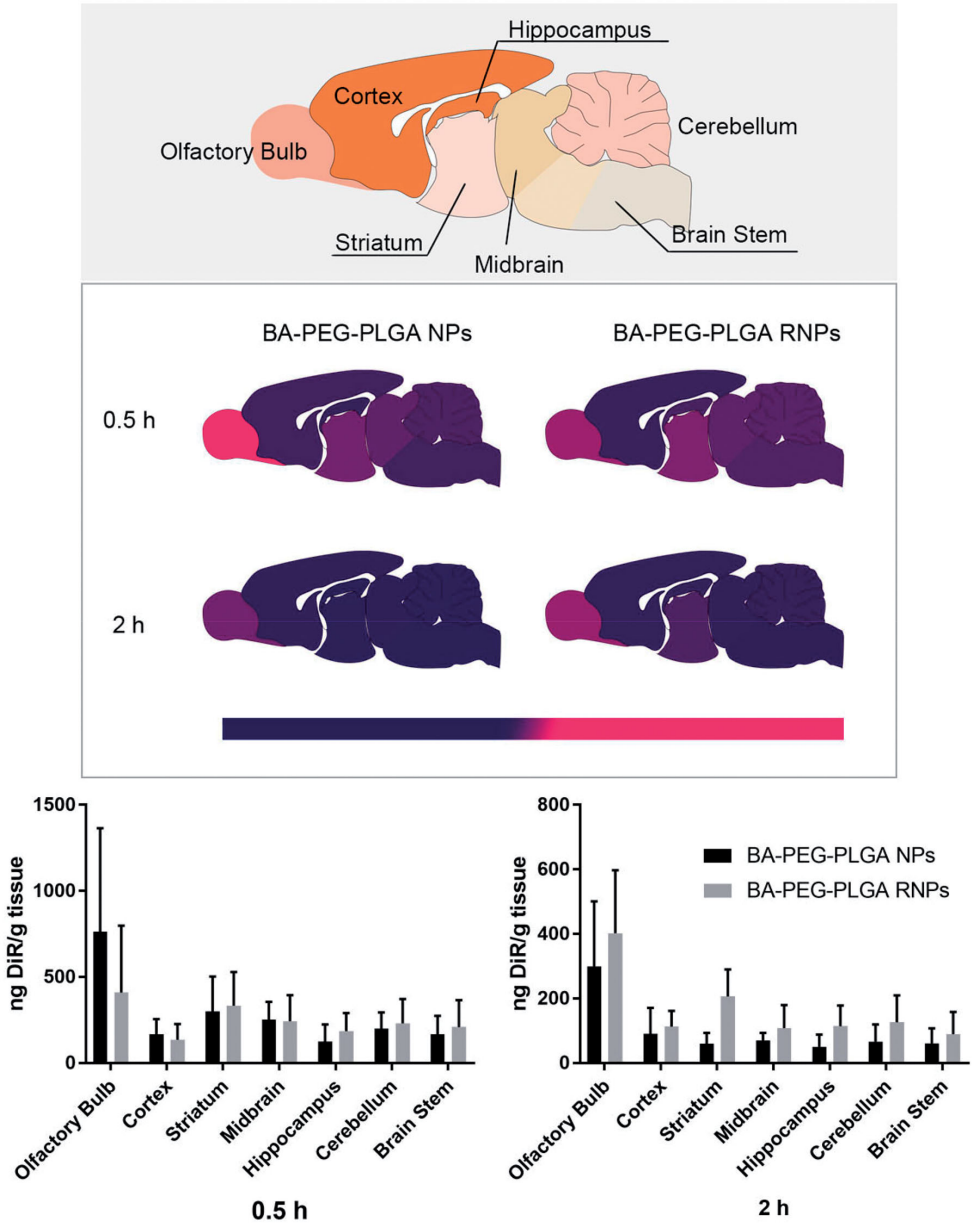
Biodistribution of DiR-loaded PEG–PLGA NPs and PEG–PLGA RNPs after intranasal administration. The figure shows a sagittal schematic diagram of brain regions. The graphs show the mean ± SD (*n* = 5).

Figure 4 shows the biodistribution of PEG-PLGA NPs and PEG-PLGA RNPs 2 h after intranasal delivery of the particles. DiR delivered by both types of NPs showed the highest concentration in the olfactory bulb. RNPs had delivered higher amounts of DiR to the whole brain than had PEG-PLGA NPs 2 h after intranasal administration (*p* > .05).

### BA preparations reduce cerebral infarct volume in rats with ischemic brain injury

**Figure 5.**
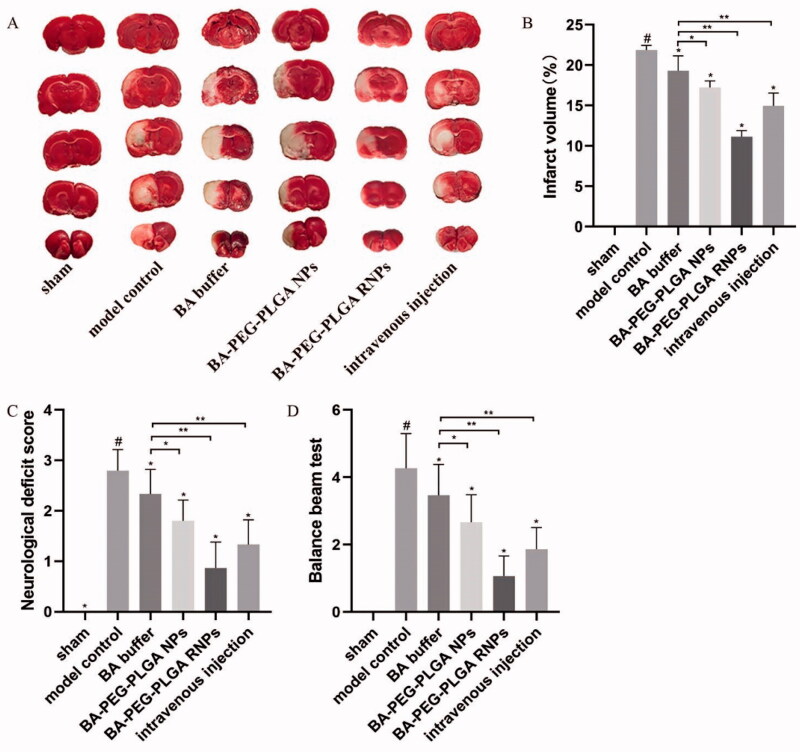
The neuroprotective effect of BA on MCAO rats in terms of cerebral infarct volume (A, B), neurological function injury (C), and recovery (D).The graphs show the mean ± SD. #*p* < .05, compared with the Sham group, *p < .05, compared with the MCAO group. Intravenous injection refers to the BA–PEG–PLGA RNP group with intravenous administration.

As shown in Figure 5(A,B), both sides of the brains of the rats in the sham-operated group appeared red after TTC staining, indicating that those rats did not suffer from cerebral infarction. Compared with the sham-operated group, significant infarct volume was observed in the model group; at the same time, the infarct volume in the model group was significantly larger than those in the animals in all of the treatment groups. Compared with the BA buffer group, the BA–PEG–PLGA NP group, and the BA–PEG–PLGA RNPs with intravenous administration group, the BA–PEG–PLGA RNPs group had the smallest infarct volume after treatment (*p* < .05). This shows that the BA preparation can effectively improve recovery from ischemic brain injury in rats.

### Effects of BA on neurological damage

The neurological function of the rats in each group was evaluated. The neurological deficit due to ischemic brain injury was smallest in the animals in the BA–PEG–PLGA RNPs group. Each treatment group was also significantly better than the model group with respect to neurological deficits (*p* < .05) (see Supplementary Material Video S1), and all of the treatments had discernible effects on neurological recovery, as shown in Figure 5(C,D). It is suggested that the nasal administration of targeted modified BA NPs has the most obvious effect on recovery from damage to nerve function.

### NP treatment attenuated the severity of nerve damage in CI rats

#### Ultrastructure of rat nerves observed by TEM

The brain samples obtained from the sham group showed clear and continuous neuronal membranes under TEM, with obvious and uniform chromatin. Plentiful and intact mitochondria, numerous ribosomes, intact endoplasmic reticulum (ER), and Golgi apparatus were observed. Obvious edema was observed in the MCAO model group, and other abnormal morphologies, such as mitochondrial swelling, vacuolization, swollen ER, and discontinuity of the nuclear membrane, were observed. After treatment with BA, the edema was relieved, but obvious swelling of the ER, fragmented mitochondria, and abnormally shaped nuclei could still be observed. After treatment with BA–PEG–PLGA NPs, the morphology of the nuclei tended to be normal, and the neurons contained a few vacuoles and swollen mitochondria. After nasal inhalation or intravenous injection of BA–PEG–PLGA RNPs, mitochondrial swelling was alleviated, but mild swelling of the endoplasmic reticulum remained in the intravenous injection group that received BA–PEG–PLGA RNPs. In the animals that received BA–PEG–PLGA RNPs by nasal inhalation, the morphology of the nuclei had returned to normal, the nuclear membrane was intact, and ribosomes and mitochondria were abundant.

#### HE staining of brain tissue sections

The neurons in the sham group displayed normal morphology with rich Nissl bodies and densely arranged nerve fibers. In the model group, the cell layers in the hippocampus were disordered; the neurons were arranged loosely, some neurons were necrotic or had disappeared, and the neuronal nuclei appeared shrunken. After treatment with BA buffer, edema was alleviated compared with the model group, but some inflammatory cell infiltration was observed. After treatment with BA–PEG–PLGA NPs, the morphology of the nuclei tended to be normal, and nerve damage was reduced. Compared with the model group, the Nissl bodies of the hippocampal neurons in the group that received BA–PEG–PLGA RNPs by intravenous injection were more obvious but not abundant. Compared with the model group, the morphology and arrangement of neurons in the hippocampus were relatively normal after nasal administration of BA–PEG–PLGA RNPs.

### Effects of BA treatment on Nrf2 and HO-1 immunoreactivity in the brains of CI rats

The immunohistochemistry results showed that the number of Nrf2-positive cells was relatively small in the sham group. In the CI model group, the number of cells with high expression of Nrf2 and HO-1 was very small. After BA treatment, compared with the model group, the number of Nrf2 and HO-1 positive immunoreactive cells increased in the BA–PEG–PLGA NPs group (*p* < .05) and the BA–PEG–PLGA RNPs group (*p* < .05) by nasal inhalation, and in the intravenously injected BA–PEG–PLGA RNPs group (*p* < .05); a few positive cells were also observed in the BA buffer group (Figures 8 and 9). The expression levels of Nrf2 and HO-1 were upregulated most significantly in the group that received BA–PEG–PLGA RNPs by nasal administration (*p* < .05), and brown cytoplasm and nuclei were seen.

### BA administration reduces inflammatory factor levels

As shown in Figure 10, the levels of the inflammatory factors IL-1β, IL-6, and TNFα were significantly increased in the model group compared with the sham operation group. While the BA–PEG–PLGA RNPs group showed significantly reduced levels of IL-1β, IL-6, and TNFα, the BA buffer group and the BA–PEG–PLGA NPs group and the BA–PEG–PLGA RNPs with intravenous administration group also showed reduced levels of the above three factors to varying degrees, and compared with the model group, there were also significant differences (*p* < 0.05). This finding indicates that BA administration can reduce the levels of inflammatory factors in rats with ischemic brain injury.

### Effect of BA preparations on oxidative stress

Administration of BA preparations can effectively reduce the oxidative stress caused by cerebral ischemia. First, compared with the model group, the BA buffer group, the BA–PEG–PLGA NPs group, the BA–PEG–PLGA RNPs group and the BA–PEG–PLGA RNPs with intravenous administration group showed increased levels of SOD and GSH and reduced levels of MDA and ROS. It can be seen that BA administration has the potential to defend against the oxidative stress induced by CI and that the modified BA NPs have the most obvious effect, as shown in Figure 11.

## Discussion

The development of brain-targeting drug delivery systems has important clinical implications. At present, the clinical administration of drugs to treat brain disease relies mainly on brain implantation and intraventricular and intracerebral injection, methods of administration that not only carry a high risk of infection but also cause significant trauma to patients (Wu et al., 2019). These disadvantages are attributed to the existence of the BBB. Developing effective drug delivery methods that allow drugs to cross the BBB is advisable for the treatment of brain diseases. Encapsulating drugs in NPs functionalized with brain-targeting ability is considered one of the most promising approaches. PEG–PLGA biomaterials have been approved as medical standard materials by the U.S. Food and Drug Administration (FDA) due to their good safety, altered molecular weight, and high biocompatibility and are widely used in the development of drug delivery systems. NPs prepared using PEG–PLGA offer the advantages of suitability for long-term administration and controlled release in the brain (Wang et al., 2021).

For brain administration, especially intranasal delivery, NPs with certain properties are needed. The NPs prepared by PEG–PLGA and used in this study were designed to have particle sizes of <120 nm. NPs with small particle sizes may be more advantageous for nasal-to-brain delivery, although a certain amount of drug encapsulation efficiency may be sacrificed due to their small size. We used two methods including emulsion-solvent evaporation and nanoprecipitation to prepare NPs. Considering the uniformity and drug-loading properties of the NPs to fit brain delivery, surfactants were moderately added. The quality of formulations containing different concentrations of surfactants was investigated. From our results, it can be seen that the N1, N2 formulations with surfactants obtained smaller average particle size than the N3, N4 formulations without surfactants. Higher concentrations of surfactant formulations (F3, F4 with 1% SC) have smaller particle sizes than those obtained with lower concentrations (F1, F2 with 0.5% SC). For example, NPs from the same PEG–PLGA prepared with different concentrations of surfactant obtained different particle sizes. The particle size of the F1 formulation was 112.5 ± 13.08 nm, while that of F3 formulation was 103.25 ± 16.35 nm. From our eight formulations, we selected the F4 and N2 formulations for in vivo testing based on the NP size, PDI, and encapsulation efficiency of each formulation, although their encapsulation efficiency was not the highest (75% and 64%, respectively). We believe that the targeting ability of NPs used for N2B is of primary importance and that the degree of ligand modification may be a vital factor; therefore, we further tested the concentrations of RVG29 in BA–PEG–PLGA RNPs prepared using the F4 and N2 formulations. The N2 formulation was finally chosen because its RVG29 concentration was higher than that of F4 (see Supplementary Material Table S1).

Effective treatment of stroke patients with medication is only achieved when the drugs are administered within a certain time window. BA has been found to significantly reduce damage following cerebral infarction and to improve neurological function when administered within a time window of 4 h, demonstrating the potential for the use of BA in the treatment of stroke (Liang et al., 2017). However, the therapeutic efficacy of BA is limited by its rapid elimination, its short half-life in plasma, and its poor solubility in water(Zhang et al., 2016). The use of drug carriers to assist *in vivo* delivery of BA often helps improve its bioavailability. A variety of methods, including the formation of nanoemulsions that improve the oral bioavailability of BA in rats (Zhao et al., 2013), nanosuspensions consisting of coprocessed nanocrystalline cellulose-sodium carboxymethyl starch (Xie et al., 2019), and solid lipid NPs (Liu et al., 2015), have been developed for BA administration. BA is insoluble in water and in common organic solvents such as acetone and dichloromethane, and this makes it difficult to prepare and transform it for use in applications. To overcome these limitations and provide ideas for the development of BA preparations for clinical application, this study adopted two methods for the preparation of NPs for drug encapsulation, emulsion-solvent evaporation and nanoprecipitation, from which we obtained different formulations. The basic parameters of the various formulations of BA prepared by different methods were preliminarily evaluated (Figures 1 and 2). NPs prepared by both methods exhibited stable and controlled release of BA within 48 h (Figure 3).

Neuroprotection has always been one of the key points in the treatment of ischemic stroke. However, the molecular mechanism involved in CI is complex, and the therapeutic effect may not be ideal (Zhang et al., 2020). Therefore, it is extremely urgent to develop effective therapeutic drugs. As mentioned in previous reports, BA liposomes can improve nerve function defect, reduce infarct volume, and effectively improve CI reperfusion conditions (Zhang et al., 2020). Whilst the researchers are studying the mechanism evaluation, they are also devoting themselves to the development of new drug formulations for better neurotherapeutic effect. The results of our study showed that the nasal delivery of modified BA-loaded NPs may play a better neuroprotective effect. Compared with the model group and the BA buffer group, the BA–PEG–PLGA RNP group can better reduce the infarct volume of stroke area, alleviate nerve defect, and promote nerve recovery in rats (Figure 5). Our results are consistent with previous studies. It also suggests that this strategy may have a potential role as an effective and feasible treatment option for cerebral ischemia.

**Figure 6.**
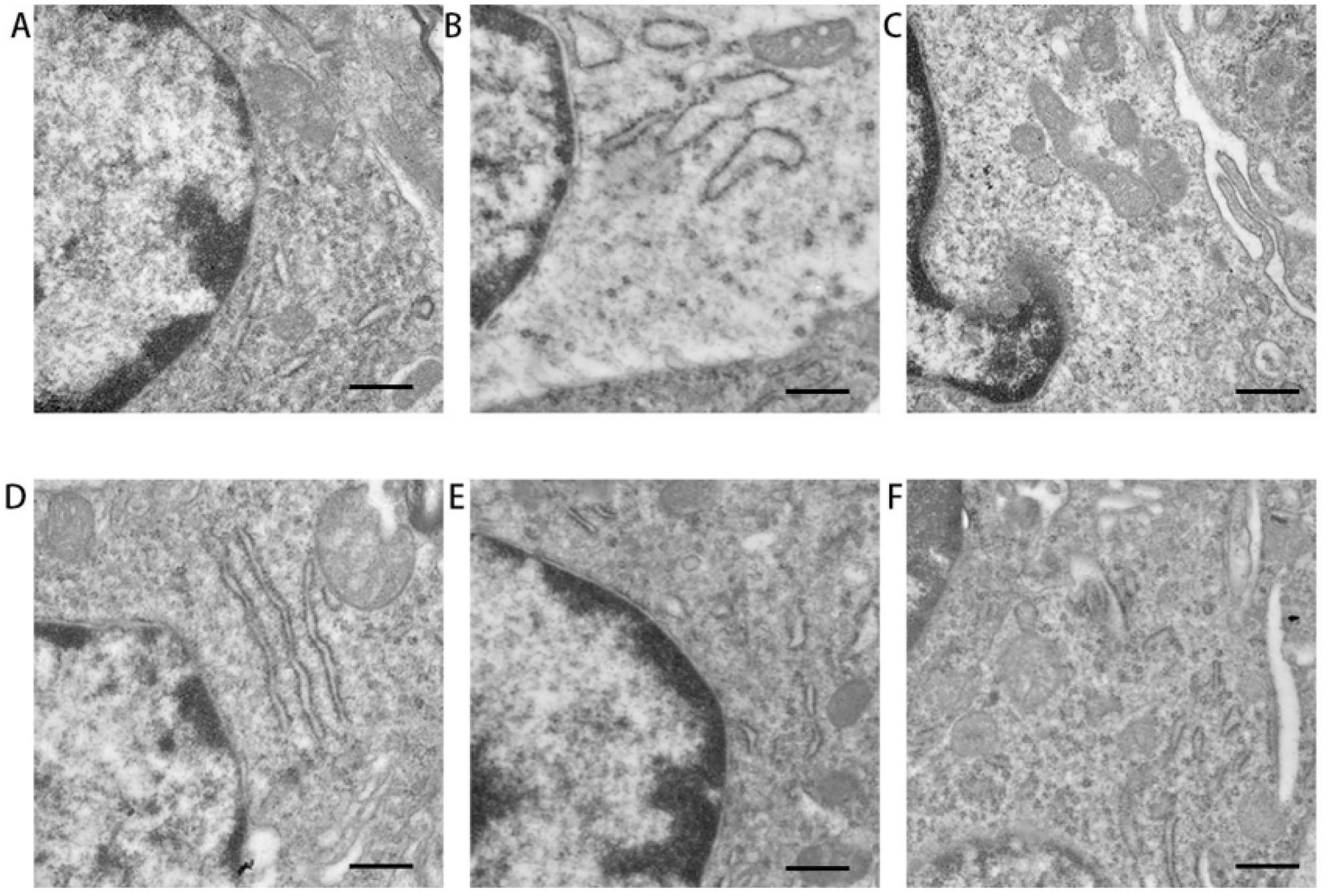
Ultrastructure of rat neurons viewed by TEM in each group. Scale bar: 500 nm. Graphs show the mean ± SD (*n* = 3). (A) sham group; (B) model control group; (C) BA buffer group; (D) BA–PEG–PLGA NP group; (E) BA–PEG–PLGA RNP group; (F) BA–PEG–PLGA RNPs with intravenous administration.

**Figure 7.**
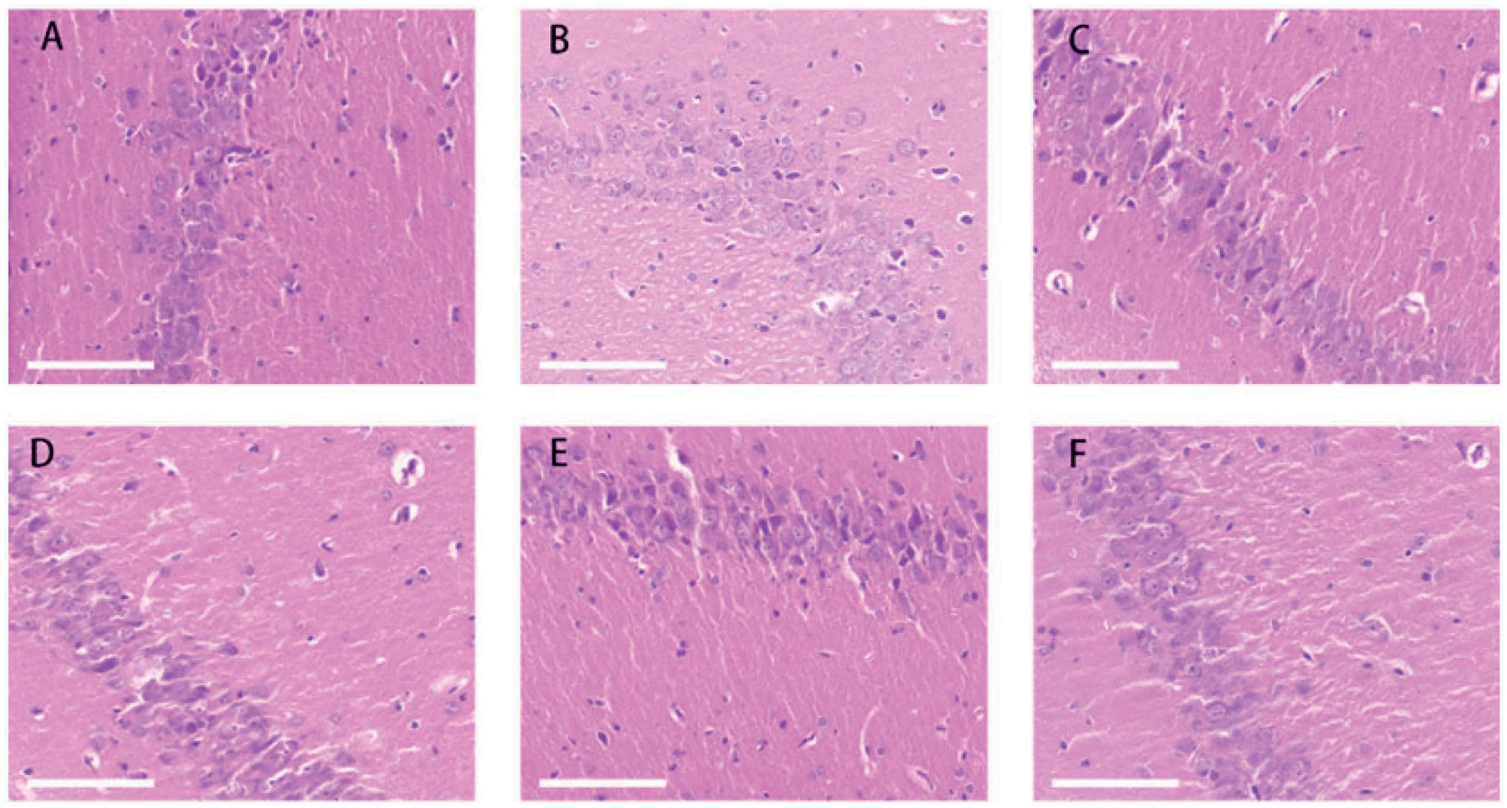
HE staining of sections of the hippocampus of rat brain (scale bar: 200 μm). Graphs show the mean ± SD (*n* = 3) (A) sham group; (B) model control group; (C) BA buffer group; (D) BA–PEG–PLGA NP group; (E) BA–PEG–PLGA RNP group with nasal administration; (F) BA–PEG–PLGA RNP group with intravenous administration.

More attention should be given to the efficiency of drug action in the brain when medication is administered. The use of intravenous injection and brain-targeting peptide-modified drug carriers may enable some drugs to cross the BBB. In the targeted therapy of some brain tumor diseases, powerful brain-penetrating peptides can be conjugated with nanomaterials and the resulting conjugates delivered to the brain via receptor-mediated transcytosis (RMT). Some peptides, such as transcriptional transactivator and cytoplasmic transducing peptide, are able to specifically bind to receptors and are then transported across the luminal membranes of endothelial cells in the BBB (Duffy et al., 1988). It should be noted, however, that opening the BBB may be associated with a risk of brain injury. Therefore, specific modifications of the carrier are important for drug delivery to the brain. In this study, we used the brain-targeting peptide RVG29, which serves as a brain-targeting ligand for the RMT-based system (Kim et al., 2013). Short RVG peptides can bind to acetylcholine receptors on neurons and may be relevant for drug delivery to nerves. Recently, advances have been made in neuroprotection against CNS diseases such as Parkinson’ s(You et al., 2018; Gan et al., 2019; Zhang et al., 2021) and Alzheimer’s (Han et al. 2020). In our study, the neuroprotective effect of BA mediated by RVG29 in CI was demonstrated. After delivery of BA–PEG–PLGA RNPs modified with RVG29, neuroedema in rats with ischemic brain injury was significantly reduced (Figure 6(e,f)). The cell morphology in the hippocampus of the brain tissue tended to be normal (Figure 6(e,f)). Compared with animals that received unmodified BA–PEG–PLGA NPs (Figures 6(d) and 7(d)) delivered by IN (Figure 7(e) and (f)), vacuolization and inflammatory cell infiltration in the neural tissue of the BA–PEG–PLGA RNPs group were significantly reduced. The differences among the experimental groups may be related to the presence of the RVG29 peptide, which displays an effective brain targeting effect and thereby exerts a good neuroprotective effect mediated by BA.

Both intranasal inhalation and intravenous drug delivery have been studied as methods for the delivery of compounds to treat brain disease. The NBDD pathway, with its special physiological conditions and the tight connection between the nasal olfactory mucosa and the olfactory bulb, can quickly transport drugs from the nasal cavity to the brain (Hoekman & Ho, 2011). This pathway is based on endocytosis by olfactory neurons and assisted transportation of the drug by nasal epithelial cells. When the drug transits the vestibule and reaches the nasal olfactory mucosa, it can reach the olfactory bulb and thereby enter the brain as a direct route (Martins et al., 2019). It is also possible for the drug to be delivered to the brainstem and other connected structures through the trigeminal pathway (Martins et al., 2019). However, there is also a potential indirect route from the nose to the brain; that is, the drug is directly absorbed by the vascular and lymphatic systems in the nasal respiratory epithelium and then transported to the systemic circulation, thereby entering the brain tissue through the BBB (Crowe et al., 2018). Notably, the indirect route may be accompanied by some disadvantages, including increased systemic exposure, short circulating half-life, and unfavorable pharmacokinetics (Bourganis et al., 2018). Special attention needs to be paid to the fate of drugs delivered via the nose. We examined the amount of the model drug DiR present in peripheral tissues after intranasal delivery of PEG–PLGA RNPs (Supplementary Figure S1). We found that only a small amount of fluorescence appeared in the spleen and lung, indicating that PEG–PLGA RNPs were more NBDD-compliant than other types of RNPs. Two hours after IN delivery of PEG–PLGA RNPs modified with RVG29, more DiR retention was observed in the olfactory bulb, striatum, midbrain, and hippocampus than was observed after delivery of unmodified PEG–PLGA NPs. This may be ascribed to the effect of the dense PEG coating on the NP surface on uptake of the particles by epithelial cells (de Oliveira et al., 2020), thereby reducing the distribution of the drug in brain tissue.

**Figure 8.**
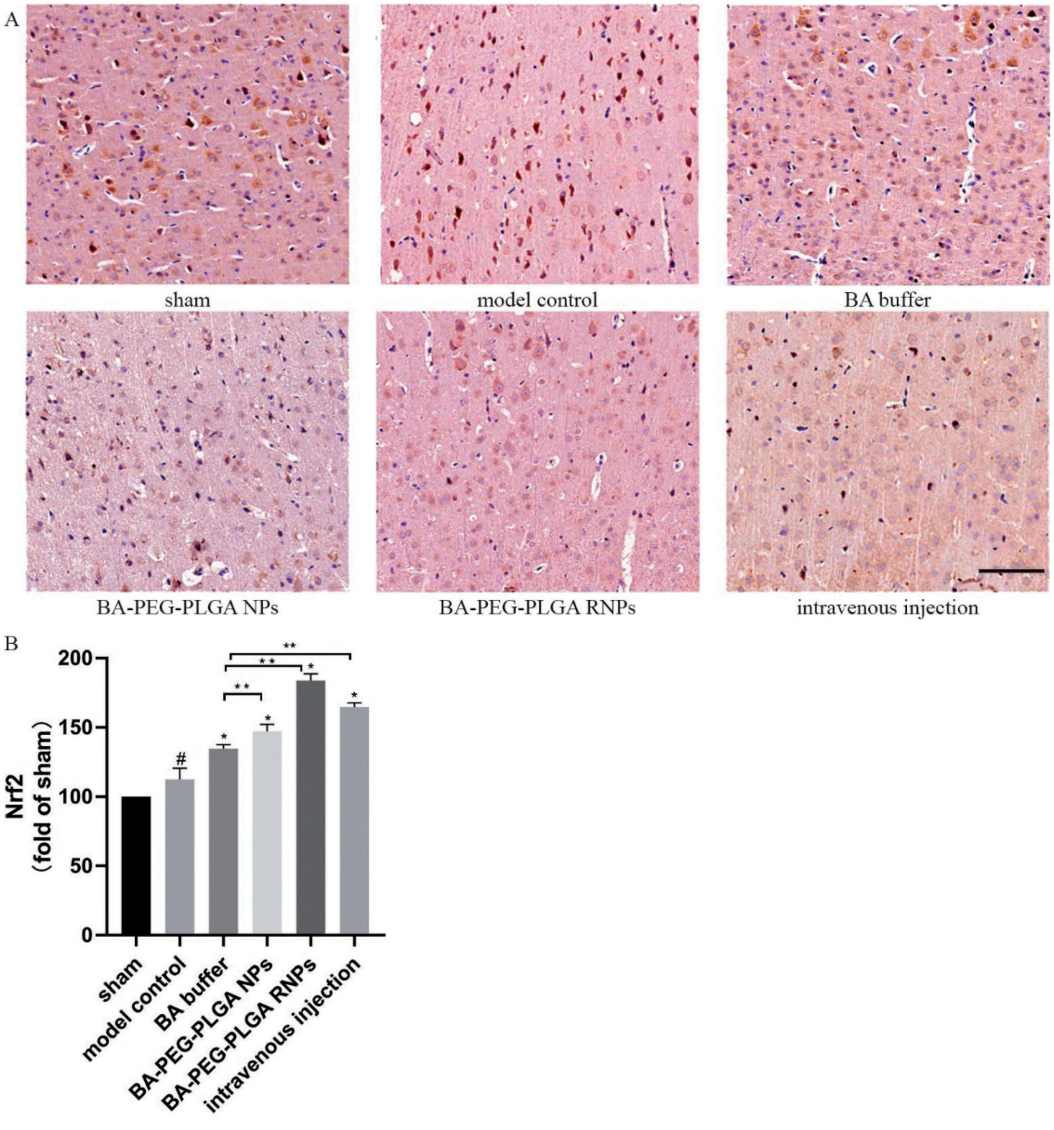
Nrf2 immunoreactivity in rat brain tissue (immunohistochemistry, light microscopy, scale bar: 100 μm). The graphs show the mean ± SD (*n* = 3). #*p* < .05, compared with the Sham group, *p < .05, compared with the MCAO group. Intravenous injection refers to the BA–PEG–PLGA RNP group with intravenous administration.

**Figure 9.**
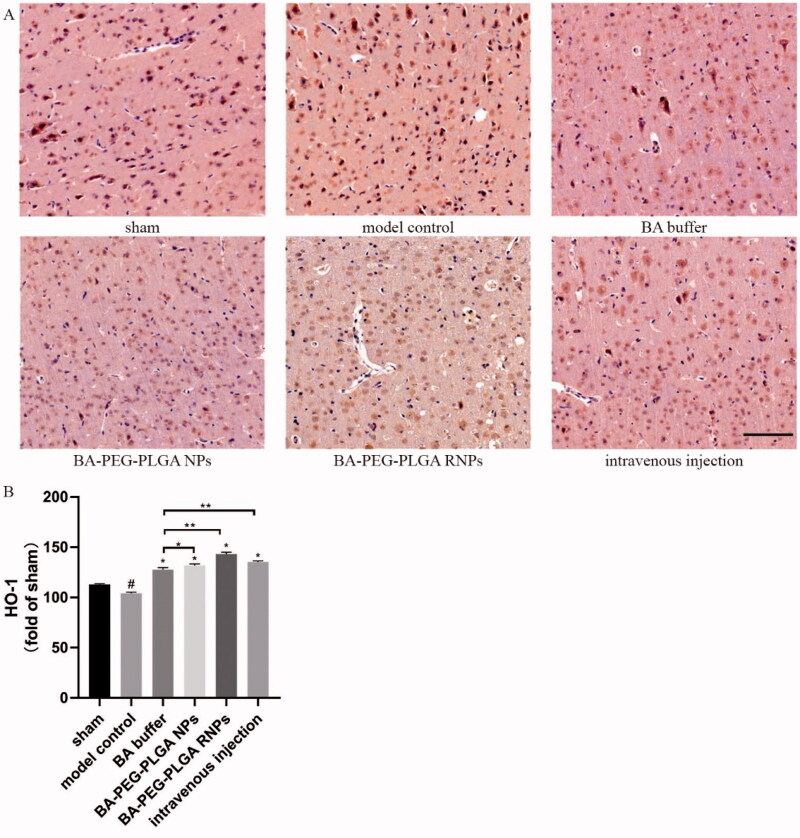
HO-1 immunoreactivity in rat brain tissue (immunohistochemistry, light microscopy, scale bar: 100 μm). The graphs show the mean ± SD (*n* = 3). #*p* < .05, compared with the Sham group, *p < .05, compared with the MCAO group. Intravenous injection refers to the BA–PEG–PLGA RNP group with intravenous administration.

Oxidative stress is an important factor that triggers poststroke injury. Nuclear factor erythroid 2-related factor 2 (Nrf2), which binds to the antioxidant response elements of genes that encode active antioxidant enzymes, is considered a major regulator of antioxidant defense responses (Shi et al., 2020). Under oxidative stress conditions, Nrf2 translocates from the cytoplasm to the nucleus and thereby plays a key role in the regulation of antioxidant defense systems by initiating the transcription of antioxidant genes. HO-1, which is regulated by Nrf2, directly affects the antioxidant balance in the body and has anti-apoptotic activity (Ya et al., 2018). In bEnd.3 cells, BA reduces the production of ROS and MDA, promotes the production of SOD, and upregulates the expression of Nrf2 and HO-1, thereby protecting against LPS-induced damage to the BBB (Wang et al., 2021). In our study, BA-loaded NPs were found to exert potent neuroprotective effects in rats suffering from ischemic brain injury. As a neuroprotective agent, BA may exert its effect through its antioxidant and anti-inflammatory properties by activating Nrf2/HO-1 (Figures 8 and 9), increasing the expression of antioxidant enzymes, alleviating oxidative stress, and reducing brain damage.

ROS are important mediators of damage in brain tissue. During ischemic stroke, excessive ROS production may not only inhibit the activity of the antioxidant enzyme SOD but may also mediate oxidative damage to DNA/RNA and ultimately induce neuronal apoptosis (Lorenzano et al., 2019). ROS are also used to assess the extent of brain cell damage (Khoshnam et al., 2017). They can disrupt lipid membranes, leading to lipid peroxidation and the production of compounds such as MDA, which can lead to protein dysfunction and increased membrane permeability. The antioxidative enzymes that work to alleviate oxidative stress mainly include GSH and SOD. SOD prevents the formation of hydroxyl radicals by catalyzing the conversion of superradicals to H_2_O_2_ (Yan et al., 2021). Accumulation of ROS and depletion of antioxidant enzymes may cause secondary brain damage (Chouchani et al., 2014). Therefore, the balance between oxidants and antioxidants needs to be controlled. The degree of lipid peroxidation that occurs after ischemic stroke can be reflected by the levels of antioxidants (SOD and GSH) and MDA (Du et al., 2020). We examined the levels of ROS, SOD, GSH, and MDA in the MCAO rat model of CI as a measure of the degree of oxidative stress in the brains of the animals. We found that oxidative stress was activated in the rat model of cerebral ischemia, the levels of ROS and MDA were significantly increased compared with those in the sham group, and the levels of SOD and GSH were decreased. However, after treatment with BA, the oxidative stress was relieved. In the group to which BA–PEG–PLGA RNPs were delivered by nasal inhalation, the levels of GSH and SOD were significantly increased, and those of ROS and MDA were significantly decreased, compared with the model group.

**Figure 10.**
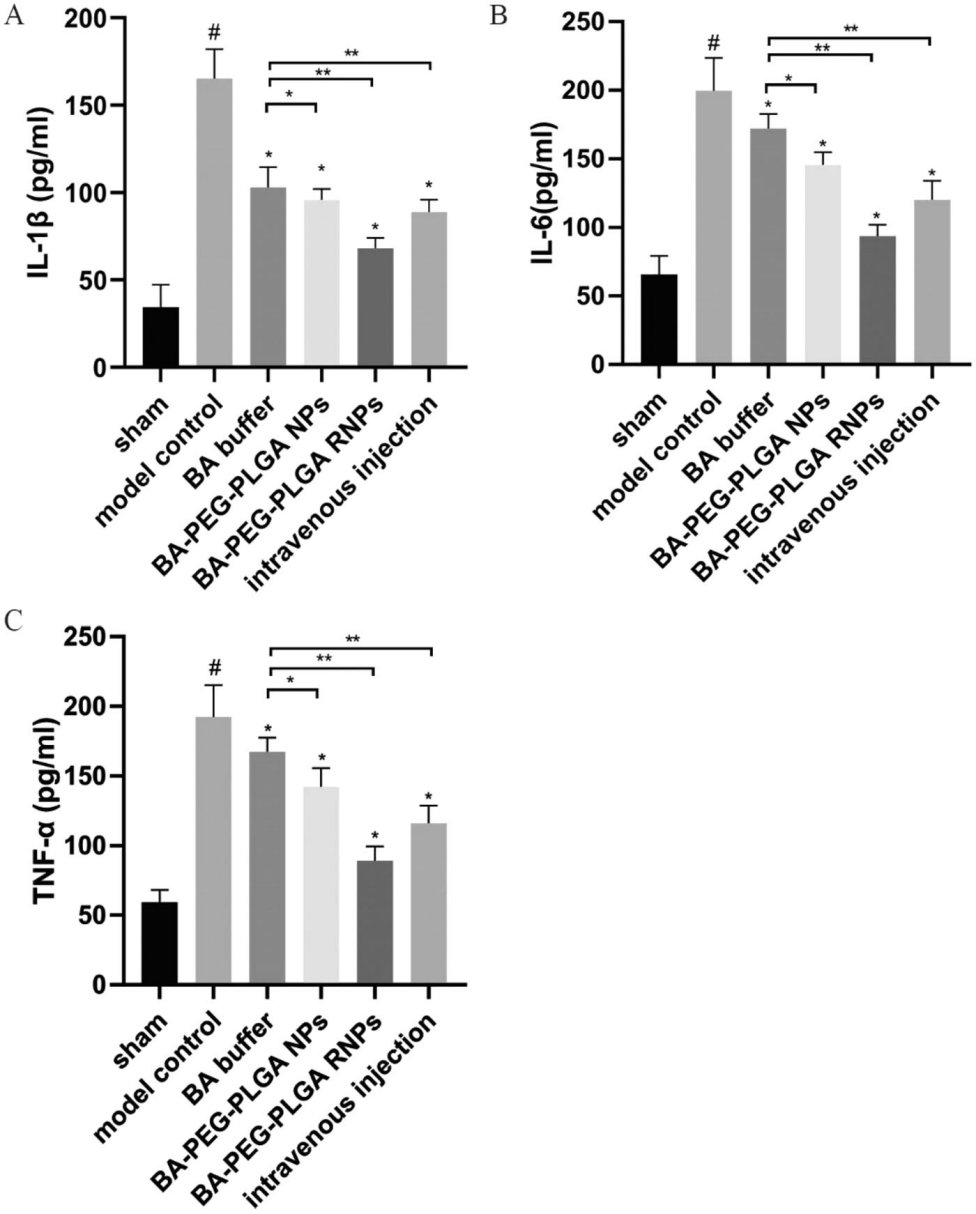
BA administration reduces the levels of inflammatory factors in rats with ischemic brain injury. ELISA was used to detect levels of IL-1β (A), IL-6 (B), and TNF-α (C). Graphs show the mean ± SD (*n* = 6). #*p* < .05, compared with the Sham group, *p < .05, compared with the MCAO group. Intravenous injection refers to the BA–PEG–PLGA RNP group with intravenous administration.

Stroke induces an acute inflammatory response characterized by elevated levels of a series of cytokines such as IL-1β, IL-6, and TNF-α in the cerebrospinal fluid (Shi et al., 2019). Inflammatory mediators may also permeate the body, causing systemic inflammatory responses. Systemic elevations of IL-6 and TNF-α have been reported to be associated with cognitive decline and stroke recurrence. It is important to monitor the inflammatory response throughout the body. In our study, we measured the levels of the inflammation-related factors IL-1β, IL-6, and TNF-α in the serum of each group of rats (Figure 10). Compared with the sham group, the model group showed a significant increase in the levels of various inflammatory factors in serum. After treatment with BA, the levels of the inflammatory-related factors IL-1β, IL-6, and TNF-α decreased compared with those in the model group (Figure 10). This may be mainly due to the anti-inflammatory properties of BA (Hu et al., 2021), which can attenuate the production of proinflammatory cytokines (e.g. IL-1β and IL-6) (Shi et al., 2017). Compared with the application of BA in buffer alone, administration of NPs carrying BA resulted in a higher level of regulation, a result that might be attributed to the fact that the NPs effectively deliver BA to the brain and thereby allow BA to better exert its anti-inflammatory effect. Among them, BA–PEG–PLGA RNPs delivered by intranasal inhalation achieved the best anti-inflammatory effect and resulted in the greatest reduction in the levels of IL-1β, IL-6, and TNF-α. This demonstrates that NPs modified with brain-targeting peptides can be used to effectively deliver drugs to rats with CI by intranasal inhalation.

**Figure 11.**
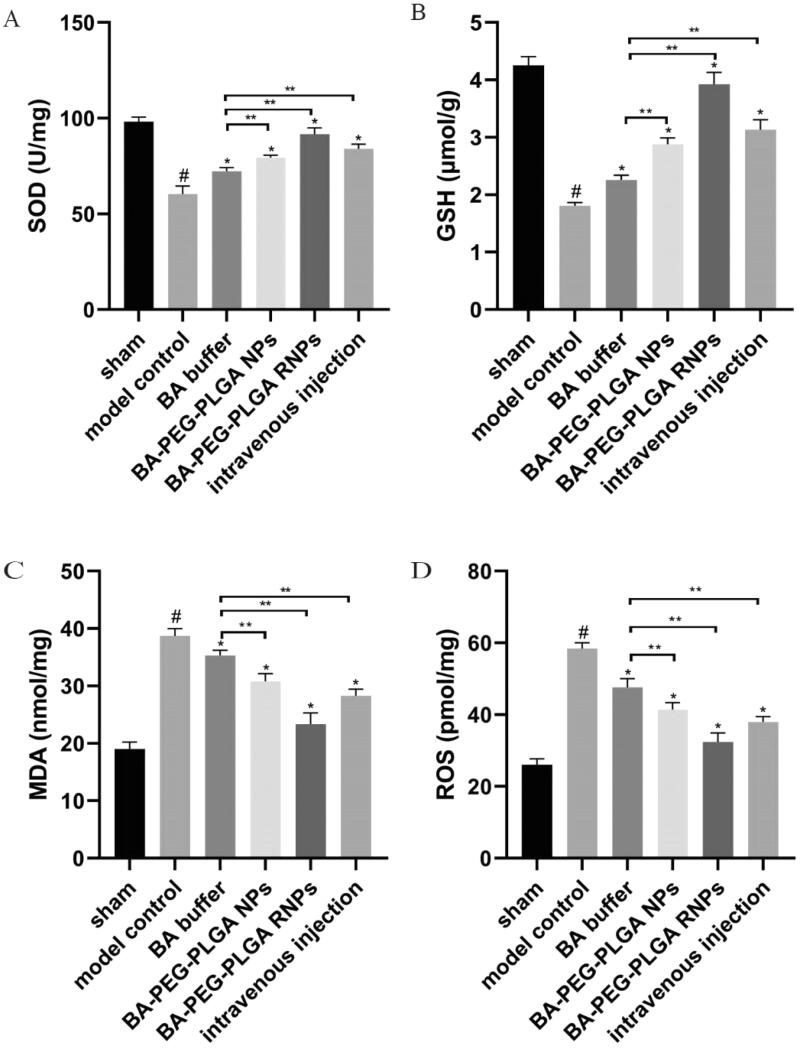
BA administration alleviates the oxidative stress caused by cerebral ischemia. ELISA was used to detect levels of SOD (A), GSH (B), MDA (C), and ROS (D). Graphs show the mean ± SD (*n* = 6). #*p* < .05, compared with the Sham group, *p < .05, compared with the MCAO group. Intravenous injection refers to the BA–PEG–PLGA RNP group with intravenous administration.

## Conclusion

We found that PEG-PLGA NPs modified with RVG29 peptide and administered through the nasal-to-brain route effectively reach the hippocampus, enabling BA to play a neuroprotective role after cerebral ischemic injury. The NPs we prepared have suitable properties as drug carriers. Neurological dysfunction in the rat model of CI was relieved after treatment with NPs, and no adverse effects on major organs were observed. This work may provide promising insight regarding clinical applications in the treatment of cerebral ischemia.

## Supplementary Material

Supplemental Material

## Data Availability

The data that support the findings of this study are available from the corresponding author upon reasonable request.
