# Supplementary material for: Preparation of baicalin-loaded ligand-modified nanoparticles for nose-to-brain delivery for neuroprotection in cerebral ischemia
Source: Drug Deliv. 2022 Apr 25;29(1):1282–98. doi: 10.1080/10717544.2022.2064564 (PMC9045769; doi:10.1080/10717544.2022.2064564)

# Supplementary information

## Table S1. The degree of conjugating of RVG29 of different formulations.

| **Formula** | **F4** | **N2** |
| --- | --- | --- |
| **RVG29-loaded** | 3.2 μg/mg | 5.8 μg/mg |

**Table S2.** The stability testing of BA-PEG-PLGA NPs stored at -20°C.

| **Time（days）** | **EE%** |
| --- | --- |
| 0 | 64.16 |
| 7 | 64.26 |
| 14 | 64.10 |
| 28 | 63.87 |
| 60 | 64.12 |
| 90 | 59.85 |

## Figure S1. DiR concentrations were measured in peripheral organs after intranasal administration of PEG-PLGA RNPs.（n=5）


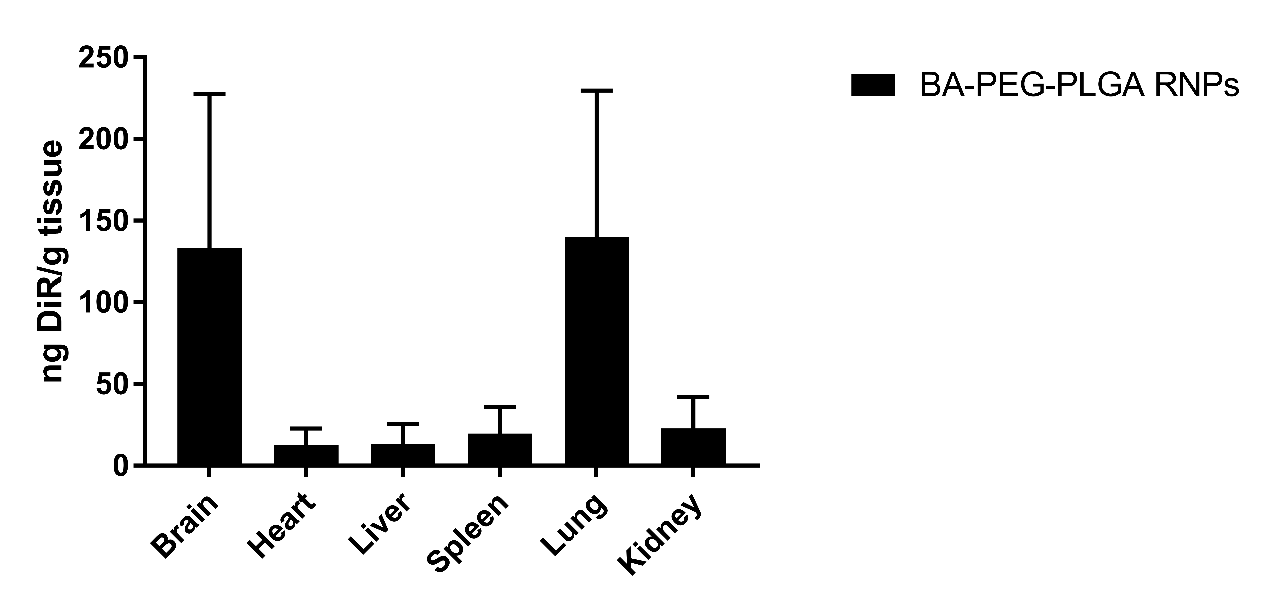

Supplement: Supplemental Material [file IDRD_A_2064564_SM4341.zip › Supplementary information.docx]
